# Standardizing Neonatal Body Composition Assessment Using Air Displacement Plethysmography: Insights from the Bavarian Experience

**DOI:** 10.3390/children12060733

**Published:** 2025-06-04

**Authors:** Lennart A. Luecke, Christoph Fusch, Gisela Adrienne Weiss, Katja Knab, Stefan Schäfer, Jasper L. Zimmermann, Anastasia Meis, Stephanie Lohmüller-Weiß, Kerstin Simon, Julia Welsch, Ursula Felderhoff-Müser, Niels Rochow

**Affiliations:** 1 Department of Anaesthesiology and Intensive Care Medicine, Campus Charité Mitte und Charité Campus Virchow-Klinikum, Charité-Universitätsmedizin, 13353 Berlin, Germany; lennart.luecke@charite.de; 2 Research Department of Child Nutrition, University Hospital of Pediatrics and Adolescent Medicine, St. Josef-Hospital, Ruhr University Bochum, 44791 Bochum, Germany; 3 Department of Pediatrics, Paracelsus Medical University, Breslauer Str. 201, 90471 Nürnberg, Germany; christoph.fusch@klinikum-nuernberg.de (C.F.); stephanie.lohmueller-weiss@klinikum-nuernberg.de (S.L.-W.); kerstin.simon@klinikum-nuernberg.de (K.S.); julia.welsch@klinikum-nuernberg.de (J.W.); 4 Department of Pediatrics, McMaster University, Hamilton, ON L8S 4L8, Canada; 5 Department of Pediatrics I, Neonatology, Pediatric Intensive Care, and Pediatric Neurology, University Hospital Essen, University of Duisburg-Essen, Hufelandstr. 55, 45147 Essen, Germany; ursula.felderhoff@uk-essen.de; 6 Department of Pediatrics, University Medicine Rostock, 18057 Rostock, Germany

**Keywords:** neonate, air displacement plethysmography, body composition, lean mass, method analysis, clinical routine, standardization

## Abstract

Background/Objectives: Body composition plays a crucial role in neurodevelopment and the long-term health of preterm and term infants. Air displacement plethysmography (ADP), especially with the PEAPOD^®^ system, is well established in research and increasingly explored in clinical practice. Building on our team’s earlier experiences, this study aimed to (1) evaluate the safety and feasibility of ADP in preterm infants, (2) identify published clinical protocols, and (3) implement and assess a standardized routine—the Bavarian Clinical Protocol (BCP). Methods: We conducted two systematic literature reviews: one on the eligibility-to-assessment rate and safety of ADP in research contexts, and a second focusing on existing clinical protocols. In addition, we retrospectively analyzed routine ADP assessments at the NICU of Nuremberg Children’s University Hospital from January 2022 to December 2024, where the BCP had been introduced. Results: The literature review included 76 studies reporting a total of 8,317 assessments without adverse events. In experimental settings, the eligibility-to-assessment rate was 41%. We identified three published clinical protocols. Following BCP implementation, 626 of 702 eligible infants (89.1%) underwent a total of 851 ADP measurements. No adverse events were observed, and repeated assessments were integrated smoothly into clinical workflows. Conclusions: ADP can be safely and effectively incorporated into neonatal routine care. The Bavarian Clinical Protocol provides a practical framework for standardized application, improves comparability across centers, and supports the clinical use of body composition data to inform individualized nutritional strategies.

## 1. Introduction

Preterm infants depend on tailored external nutrition and feeding strategies, whereas a healthy fetus in utero benefits from placental nutrition for optimal growth of organs, body mass, and brain development. With increasing survival rates and reduced neonatal morbidity, the focus has shifted toward enhancing the quality of survival [1,2]. The evidence suggests that body composition impacts the risk of chronic diseases and improves neurodevelopmental outcomes in preterm infants. Both low and excessive fat mass are linked to a greater risk of metabolic and cardiovascular diseases [3,4,5]. Greater fat-free mass has been associated with better neurodevelopment [6,7,8].

A key objective in neonatal research is to establish routine body composition monitoring and nutritional interventions to further enhance outcomes [9]. Multiple methods have been explored for body composition assessment, including anthropometry, bioelectrical impedance analysis, dual-energy X-ray absorptiometry, and air displacement plethysmography (ADP) [10,11,12].

ADP is a promising non-invasive and radiation-free approach for clinical body composition assessment, and several studies have validated its accuracy, positioning it as the gold standard for routine body composition evaluation in both preterm and full-term infants [13,14,15,16,17]. This method is a reliable tool for routine body composition evaluation in preterm and full-term infants [12]. Assessments via the PEAPOD^®^ system (COSMED, Inc., Concord, CA, USA) have been extensively described [13,14]. The method utilizes a two-compartment model—fat mass and fat-free mass—based on body density derived from measured body volume and weight. Weight is obtained via an integrated scale, whereas body volume is calculated from the displacement of air in the chamber. The model assumes a constant fat mass density and gestational age-dependent fat-free mass density. Detailed procedural information is outlined in prior studies [13,15].

While ADP has been extensively validated and widely applied in experimental settings, its integration into clinical practice remains limited to a few institutions [16,17,18]. Building upon early experimental experiences at McMaster University, our team developed a routine protocol that was successfully introduced in the neonatal intensive care unit (NICU) at Nuremberg in early 2021 [19,20]. Despite this successful transition, there remains a critical need for a standardized protocol to ensure comparable and reliable ADP application across clinical settings. Furthermore, such a protocol would facilitate the transition of ADP from primarily a research tool to a routine clinical method.

The objectives of this study are as follows:

- To systematically review the literature on the proportion of eligible preterm infants who undergo assessment (eligibility-to-assessment rate) and to evaluate the safety of ADP assessments conducted in experimental settings.
- To systematically review the literature for comparable routine clinical protocols and standard operating procedures for ADP in body composition assessments.
- To develop and utilize a standardized clinical protocol for routine ADP use in preterm and term infants: The Bavarian Clinical Protocol (BCP).

## 2. Materials and Methods

### 2.1. Literature Review (#1 and #2)

The two literature reviews (#1 and #2) followed the PRISMA (Prevention and Recovery Information System for Monitoring and Analysis) guidelines. Keyword searches were completed via PubMed and OVID MEDLINE (all) using the search terms listed below [21]. The search period was restricted to the first of January 1995 until the fifteenth of August 2024. Both review processes were performed via a stepwise approach. First, following the retrieval of the publications identified in the literature search, titles and abstracts were screened, language restrictions (limited to the English language) were applied, and duplicates were removed. In the next step, eligibility was analyzed via full-text assessment. Aims, search terms and exclusion criteria, and collected parameters for the two separate literature reviews (#1 and #2) were as follows:

(#1) Aim: To review published studies for eligibility-to-assessment rates and potential adverse events from ADP assessments in preterm infants. The “eligibility-to-assessment rate” was defined as the proportion of infants who met the inclusion criteria (eligibility) and proceeded to undergo ADP assessments (assessment). The search terms used were “body composition” AND “air displacement plethysmography” AND “preterm”. The exclusion criteria were as follows: (1) the manuscript did not contain body composition data acquired by the authors’ institution; (2) the body composition data originated from an identical cohort with publications already included; and (3) the infants’ age at body composition assessment was ≥1 year.

Subgroup analysis was performed for articles providing insights into the recruitment and eligibility process. The parameters collected were as follows: (1) no parental consent; (2) misalignment with study criteria or difficulties enrolling (e.g., study team/PEAPOD not available); (3) discharged, transferred, or lost to follow-up; (4) medically excluded; and (5) excluded for unknown reasons.

(#2) Aim: To analyze the implementation of ADP protocols in routine clinical settings at different institutions; search terms: “air displacement plethysmography” AND “clinical routine”. The exclusion criteria were as follows: (1) body composition assessments were not performed during the first year of life; (2) body composition assessments were not performed in the clinical routine, or no information was provided regarding the body composition testing procedure; (3) literature review without data from body composition assessments and no information about potential testing routine was provided; and (4) body composition assessments were not performed via the ADP method. The parameters collected were as follows: (1) eligibility criteria, (2) exclusion criteria, (3) screening for readiness of eligible infants, (4) test frequency, (5) testing time, (6) testing location, (7) personnel, (8) time requirements, and (9) clinical utility of body composition data. A comparison of the analyzed parameters was performed via Microsoft Excel and PowerPoint^®^ Office 365 (Redmond, Washington, DC, USA).

### 2.2. Retrospective Analysis of the Bavarian Clinical Protocol

A retrospective analysis of the implementation of the BCP was performed from January 2022 until December 2024 at the NICU of the Children’s Hospital at Nuremberg General Hospital, South Campus of Paracelsus Medical School Nuremberg [22,23]. Anonymized data were exported from our REDCap NICU database, which was accessed from July to December 2024. Prior to data collection, the measurement protocol was approved by our institutional review board (#SZ_D_028.21-IX-1). According to German professional regulations for physicians, this study did not require additional Ethics Committee approval because it was a quality improvement initiative, with all prior data being available on a routine basis and analyzed in an anonymized way, which was also reported according to the Standards for Quality Improvement Reporting Excellence (SQUIRE 2.0). Descriptive statistics were performed via Microsoft Excel Office 365 (Redmond, Washington, DC, USA).

## 3. Results

### 3.1. Literature Review (#1)—Air Displacement Plethysmography in Preterm Infants

**Figure 1:**
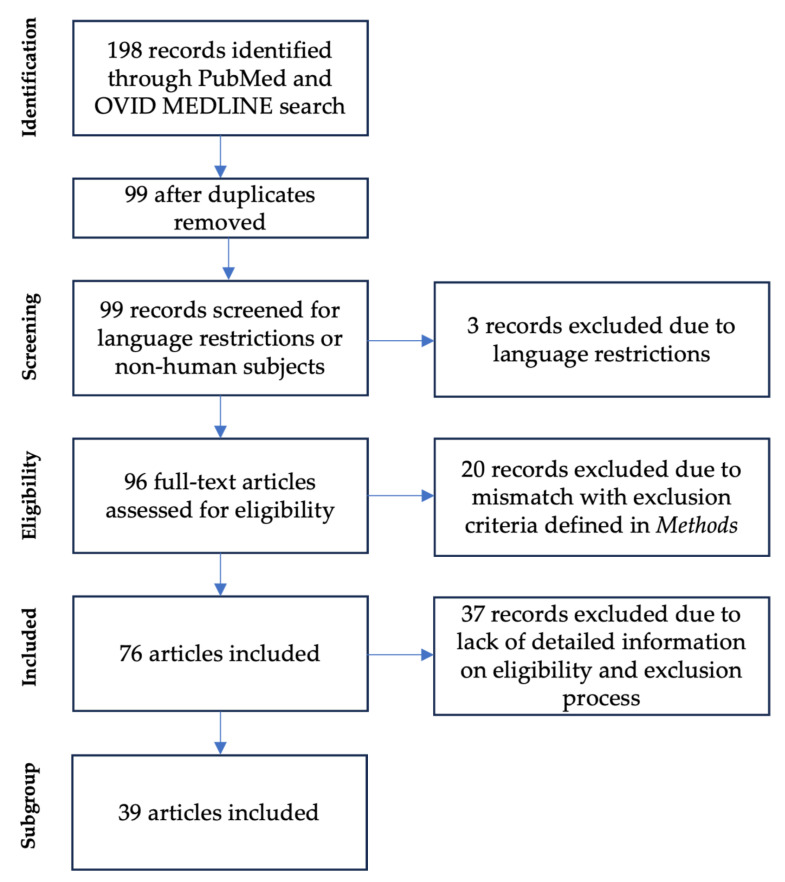
Flow chart of the literature review process #1.

A total of 198 articles were identified on the basis of the presented search terms in PubMed and Ovid. Removal of duplicates (*N* = 99) and application of language restrictions (*N* = 3) resulted in 96 articles. Eligibility was assessed with a full-text article review. *N* = 20 articles were excluded for the following reasons: (1) the manuscript did not contain body composition data acquired by the authors’ institution (*N* = 13); (2) the body composition data originated from an identical cohort with existing publications (*N* = 4); and (3) the body composition data were measured in infants older than 1 year of corrected age (*N* = 3) (Figure 1).

The resulting *N* = 76 articles provided data on body composition assessments in preterm infants. From these articles, 8317 preterm infants were tested cumulatively. Two articles were identified for full-text review for reporting potential adverse effects due to body composition measurements. These two articles reported no adverse effects due to ADP measurements.

To analyze the eligibility-to-assessment rate in an experimental setting, we performed a sub-analysis for articles providing insight into the eligibility process (manuscripts including identification of eligible infants and documentation of reasons for non-assessment) yielding *N* = 39 articles. To provide an overview of the study characteristics, eligibility criteria, exclusion criteria, and reported outcomes, a detailed summary table is available as Supplementary Table S1 [6,16,19,24,25,26,27,28,29,30,31,32,33,34,35,36,37,38,39,40,41,42,43,44,45,46,47,48,49,50,51,52,53,54,55,56,57,58,59]. These *N* = 39 articles describe a total of 8256 infants eligible for body composition measurement. Among these 8256 eligible infants, 3353 infants were enrolled and tested, resulting in a total eligibility-to-assessment rate of 40.6%. The reasons for exclusion were as follows: (1) no parental consent (*N* = 1932, 23.4%); (2) misalignment with study criteria or difficulties enrolling (e.g., study team or PEAPOD not available) (*N* = 1393, 16.8%); (3) deceased, transferred, or lost to follow-up (*N* = 794, 9.6%); (4) medically excluded (*N* = 709, 8.6%); and (5) excluded for unknown reasons (*N* = 74, 0.9%).

### 3.2. Literature Review (#2)—Clinical Routine Protocols

**Figure 2:**
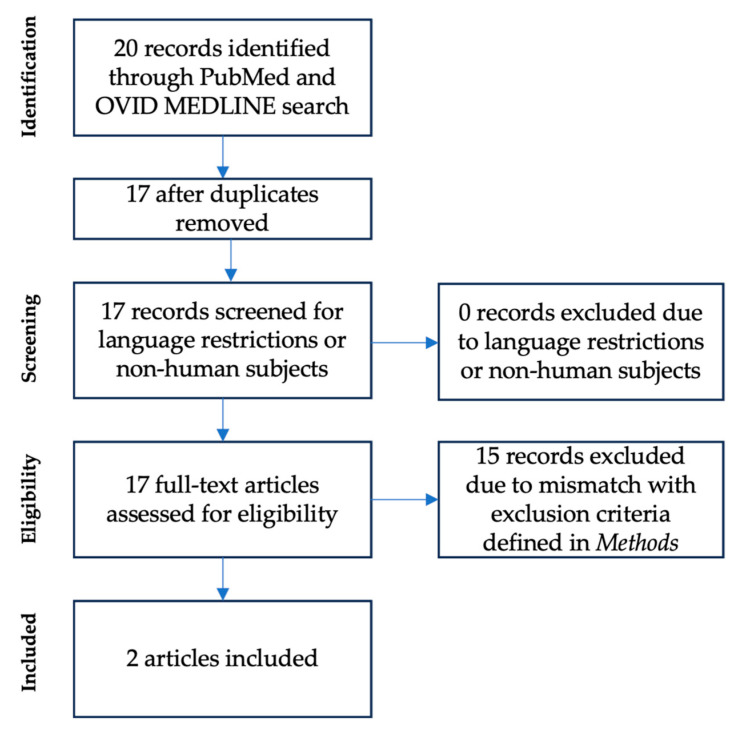
Flow chart of the literature review process #2.

A second literature review was performed to identify institutions that had already incorporated routine body composition assessments. This search initially yielded 20 articles. After removing duplicates, 17 studies remained for full-text review. Among these, 15 were excluded for the following reasons:

(1) Body composition assessments were not performed during the first year of life (corrected age, *N* = 5); (2) Body composition assessments were not performed in the clinical routine, or no information was provided regarding the body composition testing procedure (*N* = 5); (3) Literature review without data from body composition assessments and no information about potential testing routine provided (*N* = 3); (4) Body composition assessments were not performed via the ADP method (*N* = 2) (Figure 2).

The remaining two articles provided information on the setting and conditions of ADP assessments in clinical routine while comparing the protocols for implementation of the method at three pediatric hospitals: (1) Cincinnati Children’s Medical Center (Cincinnati, OH, USA), (2) MetroHealth Medical Center (Cleveland University, Cleveland, OH, USA) [17], and (3) Nuremberg Children’s University Hospital [18].

### 3.3. Comparison of Clinical Routines

The feasibility of integrating ADP into routine clinical practice was supported by studies at these three institutions. While there was general alignment of the testing protocols, several differences in practice were identified (Table 1):

1. Eligibility and exclusion criteria: Clinical stability is a common prerequisite for all three institutions. Definitions varied slightly: for example, Cincinnati Children’s Hospital excluded infants with tubes deemed critical by the surgical team, whereas at MetroHealth Medical Center, the exclusion criteria was a birth weight of >1500 g. COVID-19 infection was an exclusion criteria at both institutions. Nuremberg’s initial protocol emphasized clinical stability with no bradycardia, desaturation, or respiratory instability within 48 h before testing.
2. Screening procedures: Screening was performed by dietitians at Cincinnati, integrated into electronic medical records at MetroHealth, and handled collaboratively by nurses and physicians at Nuremberg. Here, primary screening by nurses was recommended because of their frequent patient interactions, with the attending physician confirming eligibility.
3. Testing frequency: Nuremberg and Cincinnati performed weekly assessments to track body composition changes, whereas MetroHealth limited testing to a single session during the hospital stay.
4. Time and personnel requirements: ADP assessments typically require two staff members; however, involving three operators was shown to improve efficiency, reducing the assessment time from 13 to 8 min. Approximately 12 weekly assessments at Nuremberg required up to 5 h of total staff time.

**Table 1:** Comparison of settings and conditions for ADP assessments in routine clinical practice. Data adapted from Alja’nini et al. 2021 [17] and Luecke et al. 2024 [18]. NICU = Neonatal intensive care unit.

	Nuremberg Children’s University Hospital [18]	Cincinnati Children’s Medical Center [17]	MetroHealth Medical Center [17]
**Eligibility criteria**	All infants admitted to NICU	All NICU admissions regardless of birth weight	VLBW infants (birth weight < 1500 g)
**Exclusion criteria**	1—no stable breathing on room air 2—episode of significant desaturation 3—bradycardia (<60/min) requiring stimulation within the last 48 h. 4—positive for multidrug-resistant infections on routine microbiological tests (e.g., 3-MRGN, MRSA)	1—Respiratory support or Oxygen requirement 2—Chest tube to suction 3—Tubes deemed critical by surgical team 4—COVID infection	1—Birth weight > 1500 g 2—Infants being discharged on O_2_ support and failed the ‘room air challenge’ of 2 min 3—COVID infection
**Screening for readiness of eligible infants**	The PEAPOD nurse screens all neonates at the units. Eligibility for testing is evaluated using inclusion and exclusion criteria. Clinical stability is confirmed by the attending physician on test day	Neonatal dietician brings up infant readiness for testing during daily rounds	Incorporated into NICU discharge guidelines Reminders of testing eligibility incorporated into electronic medical records
**Test frequency**	Weekly, once infant is weaned to room air	Weekly, once infant is weaned to room air	Once, at term corrected gestation or prior to dischargewhichever comes first
**Testing time**	Once weekly, Tuesdays, at predefined time window between 8.30 AM and 11.00 AM	On Wednesdays, for infants with central lines test done on ‘line change’ day	Whenever infants are ready for testing
**Testing location**	In an examination room near NICU	In the NICU	In the NICU
**Personnel**	PEAPOD certified nurse handles PEAPOD and PEAPOD nurses handle the infants	Monitor technician handles the PEAPOD device and bedside nurse handles the infant	Assigned trained NICU nurses, nurse managers and dieticians
**Time requirements**	8 min with a workforce of 3 persons (see section Time and staffing requirements for estimation of total work-load)	The measurement itself only takes 5–7 min and includes a body mass measurement	The measurement itself only takes 5–7 min and includes a body mass measurement
**Clinical utility of Body Composition Data**	Data is trended in reference graphs and available for physicians. No nutrional intervention plan is established	Trended data is used to adjust nutritional management on weekly basis.	Adjust discharge feeding regimens Evaluate/Adjust unit’s nutritional practices

### 3.4. Bavarian Clinical Protocol

**Figure 3:**
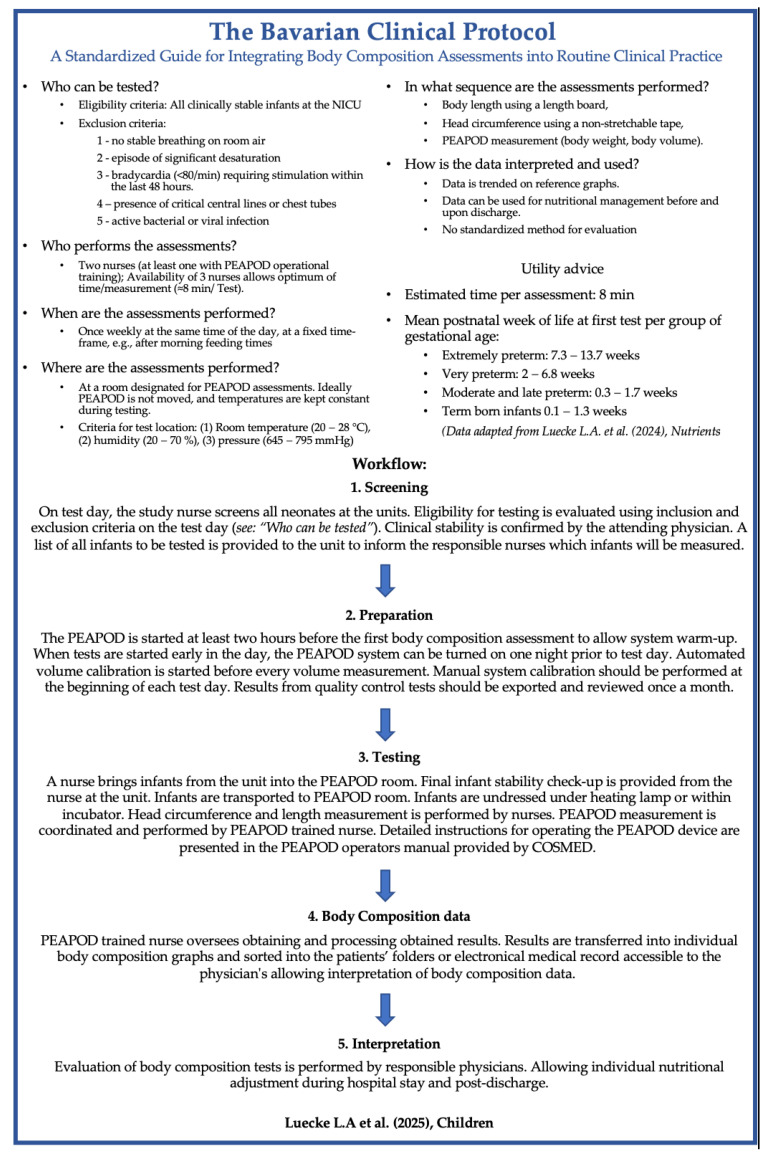
The Bavarian clinical protocol, Luecke et al. 2024 [18].

On the basis of insights from the literature review, the initial Nuremberg clinical protocol was refined into a standardized routine: the BCP (Figure 3).

This protocol was implemented in January 2022 at Nuremberg Children’s University Hospital. Between January 2022 and December 2024, 832 preterm infants were admitted to our NICU. Of these, 702 were eligible for ADP and were not receiving respiratory support on scheduled test days. Among the eligible infants, 626 underwent at least one ADP assessment, yielding an eligibility-to-assessment rate of 89.1%. In total, 851 ADP assessments were performed, including repeated measurements.

The reasons for not having body composition measurements included early discharge of late preterm infants, scheduling conflicts with other exams on the ADP measurement day, colonization with multiple drug-resistant organisms, transfer to a local hospital, and an unstable clinical condition.

No adverse events (e.g., apnea > 20 s, desaturation < 90% SaO_2_, bradycardia < 60 bpm) were observed during or within six hours of testing. Retrospective analysis revealed no device-related infections or traceable germ transmission among NICU infants. These results validate the safety and feasibility of incorporating ADP into clinical care and support its potential for improving neonatal nutritional management.

## 4. Discussion

This study systematically reviewed research employing ADP for body composition assessment in research settings, identified published protocols used in clinical practice, and successfully developed and validated the BCP. The absence of reported adverse events highlights ADP as a viable and safe research tool for assessing body composition in preterm infants. Published protocols for routine clinical application in neonates remain limited. Furthermore, this study demonstrated that the BCP is feasible and safe for implementation in routine clinical settings.

### 4.1. Current Utilization of Routine ADP Assessments for Preterm Infants

Our initial literature review (#1) revealed a low eligibility-to-assessment rate of 41% in experimental settings. The causes for not performing ADP were a lack of parental consent (23%) and not meeting the inclusion criteria (17%). In contrast, when the ADP is integrated into routine clinical practice, the eligibility-to-assessment rate is significantly higher (89.1%) on the basis of our retrospective analysis. A similar trend was noted in our previous study, where routine testing enabled assessments of almost 90% of eligible term and preterm infants [18].

Across the 79 reviewed publications, ADP was consistently described as a safe, non-invasive method for neonatal body composition assessment. Only two manuscripts addressed the possible occurrence of adverse effects related to ADP: (1) Roggero et al. reported crying infants during assessments [60], and (2) Pereira-da-silva et al. reported that “No death occurred during or close to the study period” [61]. These findings align with findings from our recent quality improvement analysis [18] and the lack of adverse events related to 851 ADP assessments over the two-year evaluation period of this current study. The safety of ADP is comparable to that of routine anthropometric measurements and provides additional insights into nutritional status and growth.

Routine ADP use has the potential to enhance neonatal care by significantly increasing eligibility-to-assessment rates while enabling safe, inclusive, and comprehensive body composition assessments for preterm and term infants with minimal practical constraints.

### 4.2. Comparison of Protocols for Routine Clinical ADP

The feasibility of routine ADP assessments has been demonstrated across institutions, including Cincinnati Children’s Medical Center, MetroHealth Medical Center, and Nuremberg Children’s University Hospital. While protocols are broadly comparable, minor procedural variations warrant discussion:

1. Eligibility criteria: Most centers exclude infants with active infections, central lines, or critical tubes, prioritizing safety and preventing cross-contamination. Formalizing these criteria across institutions may further reduce risks [62].
2. Testing frequency: Weekly assessments, as supported by our prior findings, offer sufficient reproducibility for monitoring changes in body composition. Institutions may opt for single or repeated assessments on the basis of priorities—while repeated tests improve monitoring, single assessments reduce workload [14].
3. Time and Staffing Requirements: Streamlined workflows with at least two nurses significantly reduce assessment duration, from an average of 13 min to approximately 8 min [18]. This is slightly longer than the 5–7 min reported by Alja’nini et al.; although, it is unclear if their estimate included tasks such as dressing and undressing the infant or transporting them to and from the PEAPOD room. Furthermore, the number of personnel involved in their assessments was not specified, making it difficult to directly compare total work time across these two studies [17]. Efficient scheduling, including preparing one infant while another is being assessed, further enhances time management.

### 4.3. Bavarian Clinical Protocol (BCP)

The BCP (Figure 3) was employed in 851 assessments, demonstrating its potential for the following:

1. Serve as a practical blueprint for implementing body composition testing in various clinical settings.
2. Enhance the reliability and comparability of ADP-based measurements across institutions.
3. Improve neonatal safety by standardizing exclusion criteria and operational workflows.
4. Facilitate multicenter studies and broader benchmarking efforts in neonatal care.

The protocol’s adoption in routine practice can improve the quality of body composition assessments and provide insights for optimizing nutritional management.

### 4.4. Implementing ADP in Routine Neonatal Care: Practical Considerations

On the basis of our experience, we recommend the following strategies to ease the integration of ADP into clinical practice:

1. Weekly planning: Initiate planning sessions at the beginning of each week to schedule assessments and ensure staff alignment.
2. Morning Setup: Perform early setup of the ADP device on the test day to minimize delays and interruptions.
3. Fixed Location: Designate a permanent location for the device to reduce transport time and eliminate frequent recalibration needs.
4. Streamlined Workflow: Adopt an assembly line model, with assigned roles for measurement, infant transport, and dressing/undressing.
5. Comprehensive Growth Monitoring: Pair ADP assessments with weekly anthropometric measurements to track overall growth and optimize resource allocation.

### 4.5. Interpretation and Use of Body Composition Data

Currently, there are no established guidelines for body-composition-based nutritional interventions in neonates. At the NICU of Nuremberg Children’s Hospital, longitudinal ADP measurements are digitally tracked and visualized using gestational age-specific percentile trajectories for fat mass and fat-free mass. This standardized tracking enables clinicians to identify deviations from expected growth patterns and potential nutritional inadequacies. While no direct changes in nutritional management are yet based solely on these measurements, the data support clinical decision-making and highlight the need for future evidence-based recommendations to translate body composition metrics into targeted interventions.

### 4.6. Clinical Significance

Standardized protocols such as the BCP lower the barrier to implementing ADP in neonatal care, offering an accessible approach to safe, reliable body composition assessment. Over 200 actively used PEAPOD devices globally represent a significant opportunity to safely integrate these practices into routine NICU workflows [17]. By plotting growth trajectories and utilizing reference charts (e.g., Hamatschek et al., Norris et al. and Demerath et al. [53,63,64]), clinicians can optimize nutrition and monitor individual growth patterns.

Future research should explore standardized approaches to nutritional interventions informed by body composition data. This could identify optimal compositions for supporting neurodevelopment while minimizing the risk of future metabolic or vascular conditions. The development of guidelines for data interpretation and tailored nutritional adjustments will further increase the clinical utility of routine ADP assessments.

### 4.7. Limitations

We suggest that the BCP represents a significant milestone in the nutritional management of preterm infants. However, the BCP’s applicability may vary due to differences in institutional regulations, staffing resources, logistics, and infrastructure. Additionally, the limited availability of published clinical protocols restricts direct comparisons between a high number of institutions. While this study validates the safety and feasibility of the BCP, the absence of standardized frameworks for nutritional adjustments on the basis of body composition data remains a critical gap.

The key strengths of this study are the large number of individual measurements (*N* = 851) that demonstrate the safety and feasibility of the BCP in clinical practice. To the best of our knowledge, this is the largest series of body composition assessments using ADP in a NICU setting.

## 5. Conclusions

The BCP represents a significant advance in neonatal body composition assessment, offering a robust, standardized framework for routine ADP use.

This protocol enhances scientific comparability, facilitates personalized nutrition strategies, and supports improved neonatal outcomes. Furthermore, the non-invasiveness and safety of ADP measurements are comparable to those of established anthropometric measurements. However, it provides body composition data for neonates, opening possibilities for individual nutritional adjustments on the basis of body composition reference charts.

## Data Availability

The original contributions presented in the study are included in the article, and further inquiries can be directed to the corresponding author.

## Supplementary Materials

The following supporting information can be downloaded at: https://www.mdpi.com/article/10.3390/children12060733/s1, Table S1: Subanalysis from literature review (#1): Overview of Study Characteristics of Preterm ADP Assessments.

## References

[1] Costeloe K.L., Hennessy E.M., Haider S., Stacey F., Marlow N., Draper E.S. (2012). Short term outcomes after extreme preterm birth in England: Comparison of two birth cohorts in 1995 and 2006 (the EPICure studies). BMJ.

[2] Stoll B.J., Hansen N.I., Bell E.F., Walsh M.C., Carlo W.A., Shankaran S., Laptook A.R., Sánchez P.J., Van Meurs K.P., Wyckoff M. (2015). Trends in Care Practices, Morbidity, and Mortality of Extremely Preterm Neonates, 1993–2012. JAMA.

[3] Hofman P.L., Regan F., Jackson W.E., Jefferies C., Knight D.B., Robinson E.M., Cutfield W.S. (2004). Premature birth and later insulin resistance. N. Engl. J. Med..

[4] Ong K.K., Ahmed M.L., Emmett P.M., Preece M.A., Dunger D.B. (2000). Association between postnatal catch-up growth and obesity in childhood: Prospective cohort study. BMJ.

[5] Carr H., Cnattingius S., Granath F., Ludvigsson J.F., Edstedt Bonamy A.-K. (2017). Preterm Birth and Risk of Heart Failure Up to Early Adulthood. J. Am. Coll. Cardiol..

[6] Ramel S.E., Gray H.L., Christiansen E., Boys C., Georgieff M.K., Demerath E.W. (2016). Greater Early Gains in Fat-Free Mass, but Not Fat Mass, Are Associated with Improved Neurodevelopment at 1 Year Corrected Age for Prematurity in Very Low Birth Weight Preterm Infants. J. Pediatr..

[7] Pfister K.M., Zhang L., Miller N.C., Ingolfsland E.C., Demerath E.W., Ramel S.E. (2018). Early body composition changes are associated with neurodevelopmental and metabolic outcomes at 4 years of age in very preterm infants. Pediatr. Res..

[8] Bua J., Risso F.M., Bin M., Vallon F., Travan L., Paviotti G. (2021). Association between body composition at term equivalent age and Bayley scores at 2 years in preterm infants. J. Perinatol..

[9] Bell K.A., Ramel S.E., Robinson D.T., Wagner C.L., Scottoline B., Belfort M.B. (2022). Body composition measurement for the preterm neonate: Using a clinical utility framework to translate research tools into clinical care. J. Perinatol..

[10] Dung N.Q., Fusch G., Armbrust S., Jochum F., Fusch C. (2007). Body composition of preterm infants measured during the first months of life: Bioelectrical impedance provides insignificant additional information compared to anthropometry alone. Eur. J. Pediatr..

[11] Koo W.W.K., Walters J.C., Hockman E.M. (2004). Body Composition in Neonates: Relationship Between Measured and Derived Anthropometry with Dual-Energy X-Ray Absorptiometry Measurements. Pediatr. Res..

[12] Nagel E., Hickey M., Teigen L., Kuchnia A., Curran K., Soumekh L., Earthman C., Demerath E., Ramel S. (2020). Clinical Application of Body Composition Methods in Premature Infants. J. Parenter. Enter. Nutr..

[13] Urlando A., Dempster P., Aitkens S. (2003). A New Air Displacement Plethysmograph for the Measurement of Body Composition in Infants. Pediatr. Res..

[14] Lücke L., Fusch C., Knab K., Schäfer S., Zimmermann J.L., Felderhoff-Müser U., Meis A., Lohmüller-Weiß S., Szakacs-Fusch A., Rochow N. (2024). Reproducibility of Air Displacement Plethysmography in Term and Preterm Infants—A Study to Enhance Body Composition Analysis in Clinical Routine. Nutrients.

[15] COSMED PEA POD® (2019). Infant Body Composition System Operator’s Manual.

[16] Salas A.A., Jerome M.L., Chandler-Laney P., Ambalavanan N., Carlo W.A. (2020). Serial assessment of fat and fat-free mass accretion in very preterm infants: A randomized trial. Pediatr. Res..

[17] Alja’nini Z., McNelis K.M., Viswanathan S., Goddard G.R., Merlino-Barr S., Collin M., Groh-Wargo S. (2021). Infant body composition assessment in the neonatal intensive care unit (NICU) using air displacement plethysmography: Strategies for implementation into clinical workflow. Clin. Nutr. ESPEN.

[18] Lücke L.A., Rochow N., Knab K., Schäfer S., Zimmermann J.L., Meis A., Lohmüller-Weiß S., Szakacs-Fusch A., Felderhoff-Müser U., Fusch C. (2024). Body Composition Analysis of the Clinical Routine Using Air Displacement Plethysmography: Age-Group-Specific Feasibility Analysis among Preterm Infants. Nutrients.

[19] Fusch S., Fusch G., Yousuf E.I., Rochow M., So H.Y., Fusch C., Rochow N. (2021). Individualized Target Fortification of Breast Milk: Optimizing Macronutrient Content Using Different Fortifiers and Approaches. Front. Nutr..

[20] Rochow N., Fusch G., Ali A., Bhatia A., So H.Y., Iskander R., Chessell L., el Helou S., Fusch C. (2021). Individualized target fortification of breast milk with protein, carbohydrates, and fat for preterm infants: A double-blind randomized controlled trial. Clin. Nutr..

[21] Page M.J., McKenzie J.E., Bossuyt P.M., Boutron I., Hoffmann T.C., Mulrow C.D., Shamseer L., Tetzlaff J.M., Akl E.A., Brennan S.E. (2021). The PRISMA 2020 statement: An updated guideline for reporting systematic reviews. BMJ.

[22] Harris P.A., Taylor R., Minor B.L., Elliott V., Fernandez M., O’Neal L., McLeod L., Delacqua G., Delacqua F., Kirby J. (2019). The REDCap consortium: Building an international community of software platform partners. J. Biomed. Inform..

[23] Harris P.A., Taylor R., Thielke R., Payne J., Gonzalez N., Conde J.G. (2009). Research electronic data capture (REDCap)—A metadata-driven methodology and workflow process for providing translational research informatics support. J. Biomed. Inform..

[24] Yumani D.F.J., de Jongh D., Lafeber H.N., van Weissenbruch M.M. (2021). A comparative study using dual-energy X-ray absorptiometry, air displacement plethysmography, and skinfolds to assess fat mass in preterms at term equivalent age. Eur. J. Pediatr..

[25] Gianni M.L., Roggero P., Taroni F., Liotto N., Piemontese P., Mosca F. (2009). Adiposity in small for gestational age preterm infants assessed at term equivalent age. Arch. Dis. Child. Fetal Neonatal Ed..

[26] Binder C., Buchmayer J., Thajer A., Giordano V., Schmidbauer V., Harreiter K., Klebermass-Schrehof K., Berger A., Goeral K. (2021). Association between Fat-Free Mass and Brain Size in Extremely Preterm Infants. Nutrients.

[27] McGee M., Unger S., Hamilton J., Birken C.S., Pausova Z., Kiss A., Bando N., O’Connor D.L. (2020). Associations between Diet Quality and Body Composition in Young Children Born with Very Low Body Weight. J. Nutr..

[28] Bell K.A., Matthews L.G., Cherkerzian S., Prohl A.K., Warfield S.K., Inder T.E., Onishi S., Belfort M.B. (2022). Associations of body composition with regional brain volumes and white matter microstructure in very preterm infants. Arch. Dis. Child.-Fetal Neonatal Ed..

[29] Bell K.A., Matthews L.G., Cherkerzian S., Palmer C., Drouin K., Pepin H.L., Ellard D., Inder T.E., Ramel S.E., Belfort M.B. (2019). Associations of Growth and Body Composition with Brain Size in Preterm Infants. J. Pediatr..

[30] Macedo I., Pereira-da-Silva L., Cardoso M. (2018). Associations of Measured Protein and Energy Intakes with Growth and Adiposity in Human Milk-Fed Preterm Infants at Term Postmenstrual Age: A Cohort Study. Am. J. Perinatol..

[31] Lach L.E., Chetta K.E., Ruddy-Humphries A.L., Ebeling M.D., Gregoski M.J., Katikaneni L.D. (2022). Body Composition and “Catch-Up” Fat Growth in Healthy Small for Gestational Age Preterm Infants and Neurodevelopmental Outcomes. Nutrients.

[32] Ramel S.E., Gray H.L., Davern B.A., Demerath E.W. (2015). Body composition at birth in preterm infants between 30 and 36 weeks gestation. Pediatr. Obes..

[33] Olhager E., Tornqvist C. (2014). Body composition in late preterm infants in the first 10 days of life and at full term. Acta Paediatr..

[34] Wiechers C., Avellina V., Luger B., Bockmann K., Minarski M., Maas C., Bernhard W., Poets C.F., Franz A.R. (2022). Body Composition of Preterm Infants following Rapid Transition to Enteral Feeding. Neonatology.

[35] McNelis K., Liu C., Ehrlich S., Fields C., Fields T., Poindexter B. (2021). Body Composition of Very Low-Birth-Weight Infants Fed Fortified Human Milk: A Pilot Study. JPEN J. Parenter. Enteral Nutr..

[36] Scheurer J.M., Zhang L., Gray H.L., Weir K., Demerath E.W., Ramel S.E. (2017). Body Composition Trajectories From Infancy to Preschool in Children Born Premature Versus Full-term. J. Pediatr. Gastroenterol. Nutr..

[37] Gianni M.L., Roggero P., Piemontese P., Morlacchi L., Bracco B., Taroni F., Garavaglia E., Mosca F. (2015). Boys who are born preterm show a relative lack of fat-free mass at 5 years of age compared to their peers. Acta Paediatr..

[38] Nagel E., Hickey M., Teigen L., Kuchnia A., Holm T., Earthman C., Demerath E., Ramel S. (2021). Can Ultrasound Measures of Muscle and Adipose Tissue Thickness Predict Body Composition of Premature Infants in the Neonatal Intensive Care Unit?. JPEN J. Parenter. Enteral Nutr..

[39] Olhager E., Danielsson I., Sauklyte U., Tornqvist C. (2022). Different feeding regimens were not associated with variation in body composition in preterm infants. J. Matern.-Fetal Neonatal Med..

[40] Scheurer J.M., Gray H.L., Demerath E.W., Rao R., Ramel S.E. (2016). Diminished growth and lower adiposity in hyperglycemic very low birth weight neonates at 4 months corrected age. J. Perinatol..

[41] Gianni M.L., Consonni D., Liotto N., Roggero P., Morlacchi L., Piemontese P., Menis C., Mosca F. (2016). Does Human Milk Modulate Body Composition in Late Preterm Infants at Term-Corrected Age?. Nutrients.

[42] Beunders V.A.A., Roelants J.A., Hulst J.M., Rizopoulos D., Hokken-Koelega A.C.S., Neelis E.G., de Fluiter K.S., Jaddoe V.W.V., Reiss I.K.M., Joosten K.F.M. (2021). Early weight gain trajectories and body composition in infancy in infants born very preterm. Pediatr. Obes..

[43] Calek E., Binder J., Palmrich P., Eibensteiner F., Thajer A., Kainz T., Harreiter K., Berger A., Binder C. (2023). Effects of Intrauterine Growth Restriction (IUGR) on Growth and Body Composition Compared to Constitutionally Small Infants. Nutrients.

[44] Lima P.A.T., Meio M.D.B.B., Moreira M.E.L., de Abranches A.D., Milanesi B.G., Gomes Junior S.C.S. (2022). Energy expenditure and body composition in infants with bronchopulmonary dysplasia at term age. Eur. J. Pediatr..

[45] Perrone M., Menis C., Piemontese P., Tabasso C., Mallardi D., Orsi A., Amato O., Liotto N., Roggero P., Mosca F. (2021). Energy Expenditure, Protein Oxidation and Body Composition in a Cohort of Very Low Birth Weight Infants. Nutrients.

[46] Atchley C.B., Cloud A., Thompson D., Blunt M.H., Satnes K.J., Szyld E., Ernst K.D. (2019). Enhanced Protein Diet for Preterm Infants: A Prospective, Randomized, Double-blind, Controlled Trial. J. Pediatr. Gastroenterol. Nutr..

[47] Bruckner M., Khan Z., Binder C., Morris N., Windisch B., Holasek S., Urlesberger B. (2020). Extremely Preterm Infants Have a Higher Fat Mass Percentage in Comparison to Very Preterm Infants at Term-Equivalent Age. Front. Pediatr..

[48] McLeod G., Simmer K., Sherriff J., Nathan E., Geddes D., Hartmann P. (2015). Feasibility study: Assessing the influence of macronutrient intakes on preterm body composition, using air displacement plethysmography. J. Paediatr. Child Health.

[49] Da Silva Martins A., Barbosa Baker Meio M.D., Gomes S.C.S., Lima P.A.T., Milanesi B.G., Moreira M.E.L. (2018). Growth and body composition in preterm newborns with bronchopulmonary dysplasia: A cohort study. J. Perinat. Med..

[50] Van de Lagemaat M., Ruys C.A., Muts J., Finken M.J., Rotteveel J., van Goudoever J.B., Lafeber H.N., van den Akker C.H., Schrijver-Levie N.S., Boonstra V. (2024). Growth and body composition of infants born moderate-to-late preterm fed a protein- and mineral-enriched postdischarge formula compared with a standard term formula until 6 months corrected age, a randomized controlled trial. Am. J. Clin. Nutr..

[51] Villela L.D., Meio M.D.B.B., de Matos Fonseca V., de Abranches A.D., Junior S.-C.G., da Costa A.C.C., Murta M.M., Nehab S.R.G., Soares F.V.M., Moreira M.E.L. (2018). Growth and body composition of preterm infants less than or equal to 32 weeks: Cohort study. Early Hum. Dev..

[52] Ong M.L., Cherkerzian S., Bell K.A., Berger P.K., Furst A., Sejane K., Bode L., Belfort M.B. (2024). Human Milk Oligosaccharides, Growth, and Body Composition in Very Preterm Infants. Nutrients.

[53] Demerath E.W., Johnson W., Davern B.A., Anderson C.G., Shenberger J.S., Misra S., Ramel S.E. (2017). New body composition reference charts for preterm infants. Am. J. Clin. Nutr..

[54] Salas A.A., Travers C.P., Jerome M.L., Chandler-Laney P., Carlo W.A. (2021). Percent Body Fat Content Measured by Plethysmography in Infants Randomized to High- or Usual-Volume Feeding after Very Preterm Birth. J. Pediatr..

[55] Meyers J.M., Greecher C.P., Shaffer M.L., Shenberger J.S. (2013). Potential influence of total parenteral nutrition on body composition at discharge in preterm infants. J. Matern.-Fetal Neonatal Med..

[56] Morris E.E., Miller N.C., Haapala J.L., Georgieff M.K., Ramel S.E. (2023). Preterm infant body composition, working memory, and temperament. Infant Behav. Dev..

[57] Morlacchi L., Roggero P., Gianni M.L., Bracco B., Porri D., Battiato E., Menis C., Liotto N., Mallardi D., Mosca F. (2018). Protein use and weight-gain quality in very-low-birth-weight preterm infants fed human milk or formula. Am. J. Clin. Nutr..

[58] Roggero P., Giannì M.L., Liotto N., Taroni F., Orsi A., Amato O., Morlacchi L., Piemontese P., Agosti M., Mosca F. (2011). Rapid recovery of fat mass in small for gestational age preterm infants after term. PLoS ONE.

[59] Parat S., Raza P., Kamleh M., Super D., Groh-Wargo S. (2020). Targeted Breast Milk Fortification for Very Low Birth Weight (VLBW) Infants: Nutritional Intake, Growth Outcome and Body Composition. Nutrients.

[60] Roggero P., Gianni M.L., Amato O., Piemontese P., Morniroli D., Wong W.W., Mosca F. (2012). Evaluation of air-displacement plethysmography for body composition assessment in preterm infants. Pediatr. Res..

[61] Pereira-da-Silva L., Barradas S., Moreira A.C., Alves M., Papoila A.L., Virella D., Cordeiro-Ferreira G. (2020). Evolution of Resting Energy Expenditure, Respiratory Quotient, and Adiposity in Infants Recovering from Corrective Surgery of Major Congenital Gastrointestinal Tract Anomalies: A Cohort Study. Nutrients.

[62] Infection Control Isolation Precautions–Guidelines Library. https://www.cdc.gov/infectioncontrol/guidelines/isolation/index.html.

[63] Hamatschek C., Yousuf E.I., Möllers L.S., So H.Y., Morrison K.M., Fusch C., Rochow N. (2020). Fat and Fat-Free Mass of Preterm and Term Infants from Birth to Six Months: A Review of Current Evidence. Nutrients.

[64] Norris T., Ramel S.E., Catalano P., Caoimh C.N., Roggero P., Murray D., Fields D.A., Demerath E.W., Johnson W. (2019). New charts for the assessment of body composition, according to air-displacement plethysmography, at birth and across the first 6 month of life. Am. J. Clin. Nutr..

