# Supplementary material for: Standardizing Neonatal Body Composition Assessment Using Air Displacement Plethysmography: Insights from the Bavarian Experience
Source: Children (Basel). 2025 Jun 4;12(6):733. doi: 10.3390/children12060733 (PMC12191154; doi:10.3390/children12060733)
Supplement: Supplementary file 1 [file children-12-00733-s001.zip › children-3637930-supplementary.pdf]

children

MDPI

Supplementary Table S1: Subanalysis from literature review (#1): Overview of Study Characteristics of Preterm ADP Assessments

Authors	Year	Eligibility	Exclusion	n (eligible / assessed)	Eligibility-to-Assessment (%)	Setting
Atchley et al.	2019	Infants born <32 weeks GA, clinically stable for ADP	Higher baseline risk for abnormal body composition outcomes	345 / 36	10.4%	NICU
Bell et al.	2019	Infants born <33 weeks GA; singleton or twins	Major congenital anomalies; multiples beyond twins	122 / 62	50.8%	NICU
Bell et al.	2022	Infants born <33 weeks GA; singleton or twins	Major congenital anomalies	136 / 85	62.5%	NICU
Beunders et al.	2021	Infants born <30 weeks GA, admitted to NICU	Congenital anomalies, chromosomal disorders, skeletal dysplasia	388 / 120	30.9%	NICU
Binder et al.	2021	Infants born <28 weeks GA	Major congenital anomalies, brain malformations, skeletal dysplasia	139 / 118	84.9%	NICU
Bruckner et al.	2020	Infants born <32 weeks GA	Congenital anomalies, chromosomal/skeletal disorders	100 / 74	74%	NICU
Calek et al.	2023	Infants born <37 weeks GA	Chromosomal abnormalities, major malformations	304 / 300	98.7%	NICU
da Silva Martins et al.	2018	Infants born <32 weeks GA	Congenital malformations,	95 / 67	70.5%	NICU

 https://doi.org/10.3390/children12060733

			genetic syndromes, severe morbidities			
Demerath et al.	2017	Infants born <37 weeks GA	Congenital anomalies, syndromes affecting growth	605 / 223	36.9%	NICU
Gianni et al.	2015	Infants born <32 weeks GA, birth weight <1500 g	Major congenital anomalies	67 / 63	94%	NICU
Gianni et al.	2009	Birth weight <10th percentile for GA	Congenital diseases, chromosomal anomalies	85 / 67	78.8%	NICU
Gianni et al.	2016	Infants born 34–37 weeks GA	Congenital malformations, genetic syndromes	572 / 284	49.7%	NICU
Lach et al.	2022	VLBW infants	Not reported	92 / 57	62%	NICU + outpatient
Lima et al.	2022	Infants born <32 weeks GA	Congenital malformations, infections, chromosomal abnormalities	114 / 42	36.8%	NICU
Macedo et al.	2018	Infants born <33 weeks GA	Diagnosed genetic syndromes, congenital malformations	156 / 32	20.5%	NICU + outpatient
McGee et al.	2020	VLBW infants	Serious comorbidities, congenital malformations	840 / 158	18.8%	NICU + outpatient
McLeod et al.	2015	Infants born <33 weeks GA	Congenital abnormalities, severe illness	51 / 20	39.2%	NICU
McNelis et al.	2021	VLBW infants	Major congenital anomalies, severe morbidity	218 / 30	13.8%	NICU

Meyers et al.	2013	Infants born <35 weeks GA	Severe SGA or LGA (<3rd or >97th percentile), congenital anomalies	151 / 71	47%	NICU
Morlacchi et al.	2018	Infants born ≤32 weeks GA, birth weight <1500 g	Congenital/chromosomal anomalies, skeletal dysplasia	151 / 31	20.5%	NICU + outpatient
Morris et al.	2023	Infants born <33 weeks GA, birth weight <1500 g	Congenital anomalies affecting growth	100 / 49	49%	NICU
Nagel et al.	2021	Infants born 25–32 weeks GA	Parental consent missing	221 / 63	28.5%	NICU
Olhager et al.	2022	Infants born 23–32 weeks GA	Major congenital or chromosomal anomalies	81 / 56	69.1%	NICU + outpatient
Olhager & Tornqvist	2014	Infants born ≥32 weeks GA	Birth weight >1500 g, singleton status required	166 / 29	17.5%	NICU
Ong et al.	2024	Infants born <32 weeks GA, human milk-fed	Respiratory support needs, parental refusal	126 / 65	51.6%	NICU
Parat et al.	2020	Birth weight <1500 g	Major congenital malformations, infection	127 / 36	28.3%	NICU
Perrone et al.	2021	VLBW infants (≤1500 g)	Oxygen therapy, inadequate nutrition tolerance	206 / 50	24.3%	NICU
Ramel et al.	2015	Infants born 24–32 weeks GA	Congenital anomalies, syndromes	306 / 101	33%	NICU
Ramel et al.	2016	Infants born <1500 g, appropriate for GA	SGA <10th percentile, congenital anomalies	176 / 55	31.3%	NICU + outpatient

Rochow et al.	2021	Infants born <30 weeks GA, NICU stay ≥2 weeks	Gastrointestinal malformation, major anomalies	427 / 103	24.1%	NICU + outpatient
Roggero et al.	2011	Birth weight <1500 g, singleton	Congenital diseases, chromosomal anomalies	207 / 195	94.2%	NICU + outpatient
Salas et al.	2020	Infants born 28–32 weeks GA	GI malformations, CNS anomalies	105 / 45	42.9%	NICU + outpatient
Salas et al.	2021	Infants born <32 weeks GA	PDA, NEC, other severe morbidity	105 / 86	81.9%	NICU + outpatient
Scheurer et al.	2016	VLBW, appropriate for GA	Not appropriate for GA (<10th or >90th percentile)	176 / 56	31.8%	NICU
Scheurer et al.	2017	Infants born <35 weeks GA, appropriate for GA	Clinical diagnosis of growth restriction	27 / 20	74.1%	NICU + outpatient
van de Lagemaat et al.	2024	Infants born 32–35 weeks GA	Sepsis, respiratory or circulatory failure, severe comorbidity	184 / 144	78.3%	NICU
Villela et al.	2018	Infants born ≤32 weeks GA, birth weight ≤1500 g	Congenital diseases, genetic syndromes, malformations	78 / 66	84.6%	NICU
Wiechers et al.	2022	Infants born <32 weeks GA or birth weight <1500 g	Major congenital or chromosomal anomalies	470 / 105	22.3%	NICU
Yumani et al.	2021	Infants born 24–32 weeks GA	Major congenital anomalies, severe comorbidities	237 / 89	37.6%	NICU + outpatient

To improve clarity and uniformity, we have applied slight adjustments to the wording of ‘Eligibility Criteria’ and ‘Exclusion Criteria’ columns, including shortening and standardizing terminology across studies
